# Inferring *bona fide* transfrags in RNA-Seq derived-transcriptome assemblies of non-model organisms

**DOI:** 10.1186/s12859-015-0492-5

**Published:** 2015-02-21

**Authors:** Stanley Kimbung Mbandi, Uljana Hesse, Peter van Heusden, Alan Christoffels

**Affiliations:** South African Medical Research Council Bioinformatics Unit, South African National Bioinformatics Institute, University of the Western Cape, Bellville, South Africa

**Keywords:** Transcriptome reconstruction, Transfrags, Coding potential, Multiple domain, Annotation

## Abstract

**Background:**

*De novo* transcriptome assembly of short transcribed fragments (transfrags) produced from sequencing-by-synthesis technologies often results in redundant datasets with differing levels of unassembled, partially assembled or mis-assembled transcripts. Post-assembly processing intended to reduce redundancy typically involves reassembly or clustering of assembled sequences. However, these approaches are mostly based on common word heuristics and often create clusters of biologically unrelated sequences, resulting in loss of unique transfrags annotations and propagation of mis-assemblies.

**Results:**

Here, we propose a structured framework that consists of a few steps in pipeline architecture for *I*nferring *F*unctionally *R*elevant *A*ssembly-derived *T*ranscripts (IFRAT). IFRAT combines 1) removal of identical subsequences, 2) error tolerant CDS prediction, 3) identification of coding potential, and 4) complements BLAST with a multiple domain architecture annotation that reduces non-specific domain annotation. We demonstrate that independent of the assembler, IFRAT selects *bona fide* transfrags (with CDS and coding potential) from the transcriptome assembly of a model organism without relying on post-assembly clustering or reassembly. The robustness of IFRAT is inferred on RNA-Seq data of *Neurospora crassa* assembled using de Bruijn graph-based assemblers, in single (Trinity and Oases-25) and multiple (Oases-Merge and additive or pooled) *k*-mer modes. Single *k*-mer assemblies contained fewer transfrags compared to the multiple *k*-mer assemblies. However, Trinity identified a comparable number of predicted coding sequence and gene loci to Oases pooled assembly. IFRAT selects *bona fide* transfrags representing over 94% of cumulative BLAST-derived functional annotations of the unfiltered assemblies. Between 4-6% are lost when orphan transfrags are excluded and this represents only a tiny fraction of annotation derived from functional transference by sequence similarity. The median length of *bona fide* transfrags ranged from 1.5kb (Trinity) to 2kb (Oases), which is consistent with the average coding sequence length in fungi. The fraction of transfrags that could be associated with gene ontology terms ranged from 33-50%, which is also high for domain based annotation. We showed that unselected transfrags were mostly truncated and represent sequences from intronic, untranslated (5′ and 3′) regions and non-coding gene loci.

**Conclusions:**

IFRAT simplifies post-assembly processing providing a reference transcriptome enriched with functionally relevant assembly-derived transcripts for non-model organism.

**Electronic supplementary material:**

The online version of this article (doi:10.1186/s12859-015-0492-5) contains supplementary material, which is available to authorized users.

## Background

Whole transcriptome analysis using next generation sequencing (NGS) or sequencing-by-synthesis (SBS) technologies offers the possibility of interrogating genes and their expression en masse without knowledge of their underlying genomes. Transcriptome sequencing is often preferred over genome sequencing because of the reduced size of the sequence target space and the high functional information content [1,2]. However, sequences generated from NGS platforms are often too short to represent entire protein-coding transcripts, and genomes for reference-guided transcriptome reconstruction are rare. De Bruijn graph assemblers allow *de novo* assembly of transcripts but represent only approximate computational solutions [3]. The final assembly is one of many possibilities for which there is no universally accepted heuristic verification method; it is often highly redundant and contains mis-assemblies that are difficult to identify [4]. Post-assembly processing intended to reduce redundancy typically involves reassembly or clustering of assembled sequences. This however may lead to propagation of mis-assemblies [5] and assignment of sequences to unrelated gene clusters, resulting in loss of unique annotations [6].

The main objective of transcriptome SBS is to ascribe functional labels to assembled transcribed fragments (transfrags). This is usually done via significant sequence similarity [7] or domain signature annotations [8]. Similarity-based approaches predominantly rely on transfer of functional labels of the best BLAST hits to the sequence in question [7,9,10]*.* However, low BLAST annotation coverage is often observed, in particularly in transcriptomes of non-model organisms [11,12]. The implementation of significant BLAST hit as a proxy for functional annotation has further limitations: sequences that produce significant similarity may be functionally unrelated due to divergence [13], low complexity sequences may produce high-scoring hits but have no biological relationships [14], and functional homologs may lack sequence similarity [15]. Consequently, a first large-scale assessment of protein function shows that BLAST alone is often ineffective at predicting functional labels [16]. Domain-based annotation methods (e.g. InterProScan) appreciate only presence or absence of domains. Given that domains seldom function in isolation [17], a reliable approach should involve a method that recognises the overall domain co-occurrence architecture of the sequences under examination. Prerequisite for domain-based annotation is a reliable protein prediction method that tolerates sequencing errors and frame shifts.

**Figure 1:**
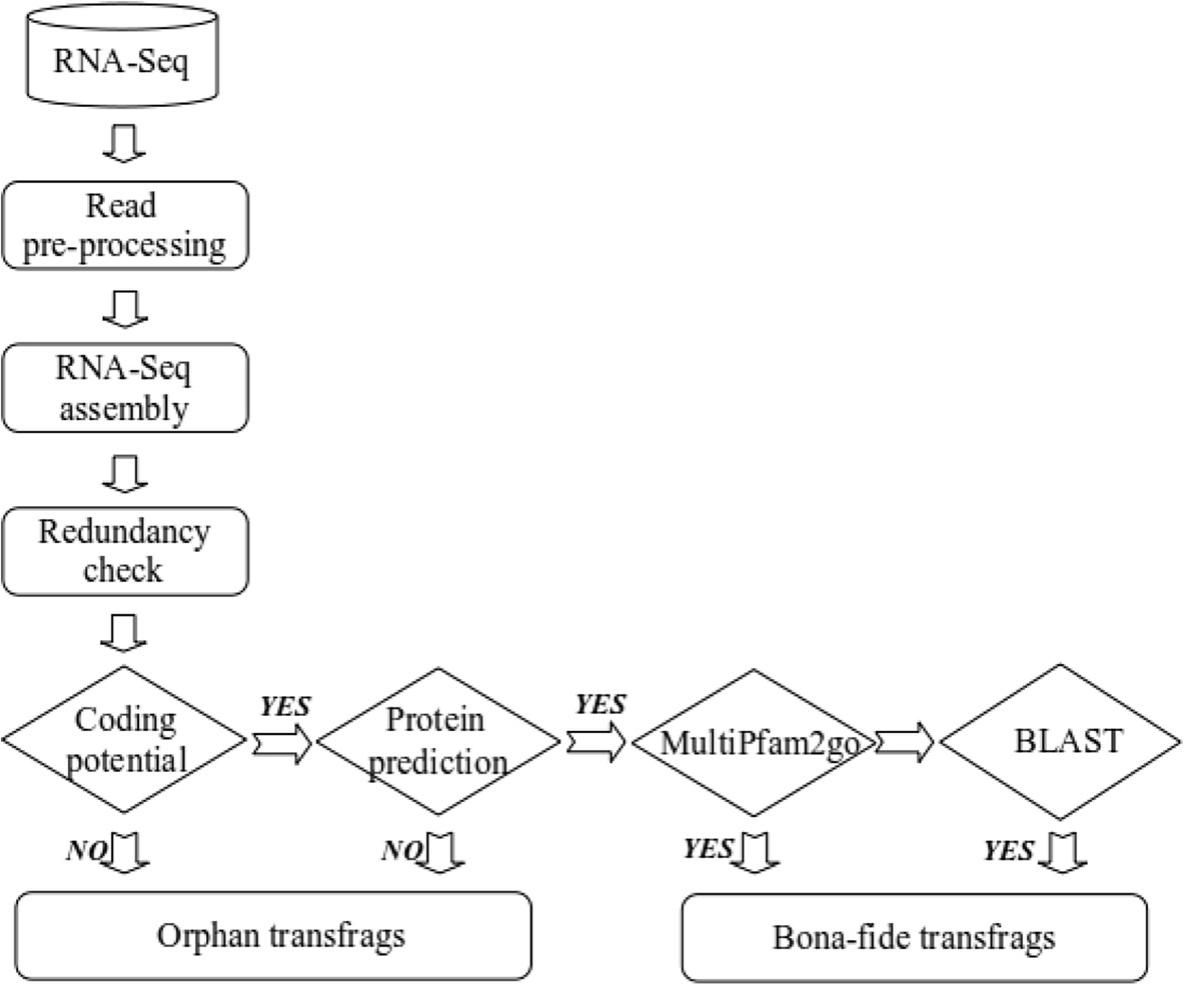
**A schematic diagram of the IFRAT pipeline.** Flow diagram to illustrate the method of integrating protein-coding potential and open reading frame prediction to infer *bona fide* assembly derived-transscripts and multiple domain co-occurrence functional annotation.

Here, we introduce IFRAT, which allows for selection and annotation of functionally relevant transfrags (*bona fide*) without clustering. This is achieved through 1) removal of identical subsequences, 2) error tolerant CDS prediction, 3) identification of coding potential, and 4) complementation of BLAST with a multiple domain architecture annotation (see Figure 1). The effectiveness and versatility of this approach is shown on published datasets from non-model organisms.

## Methods

### Availability of supporting data

To establish a robust workflow for prioritizing and selecting functionally relevant (*bona fide*) transfrags, we selected the fungal plant pathogen *Neurospora crassa* [18] as a species with a reference genome. Publicly available non-strand specific RNA-Seq data (SRR100067) from wild type *N. crassa* 74-OR23-1VA was obtained from the NCBI Sequence Read Archive (SRA, http://www.ncbi.nlm.nih.gov/Traces/sra). Untranslated 5′ and 3′ regions were procured using Ensembl BioMart [19] from http://fungi.ensembl.org release-21. The associated genomic, predicted coding sequences and Rfam family genes were obtained from the *Neurospora crassa* Sequencing Project, Broad Institute of Harvard and MIT (http://www.broadinstitute.org). We verified the pipeline using recently published transcriptomes of non-model organisms: buckwheat (*Fagopyrum esculentum*) [20]; hydra (*Hydra vulgaris*) [21]; fresh water snail (*Radix balthica*) [22]; centipede (*Alipes grandidieri*)*,* marine worm (*Cerebratulus marginatus*)*,* sea cradle (*Chiton olivaceus*)*,* mediterranean sponge (*Crella elegans*)*,* and earthworm (*Hormogaster samnitica*) [23]. The scripts, assemblies and alignment outputs generated in the ensuing analyses are available on the South Africa National Bioinformatics Institute permanent data archive (SANBI, ftp://ftp.sanbi.ac.za/ifrat).

### Read preprocessing

Quality scores of ILLUMINA reads generally depreciate towards the 3′-end. Prior to assembly, low quality bases were trimmed from the 3′-end of each sequence if above an error probability of 0.01 (PHRED base quality score of 20) using a custom PERL script with snippets from ConDeTri [24]. The quality-based filtering and trimming process ensured that orphan reads whose partner failed the quality threshold, were retained in a separate file and used for *de novo* transcriptome assembly.

### RNA-Seq assembly

Reference-free transcriptome reconstruction was performed separately using either Trinity (release 2012-06-08; [25]), or Velvet (version 1.2.03; [26]) in combination with Oases (version 0.2.06; [27]). Trinity implements greedy algorithmic traversal of the *k-*mer graph prior to building a de Bruign graph from clusters of pre-assembled sequences. As a result, assembled transfrags are represented by actual reads. Oases on the other-hand, interrogates a pre-assembly from Velvet to address alternative splicing and coverage variation across transcripts. Trinity was specifically designed for transcriptome assembly using a single, fixed *k*-mer size (*k*-25). Therefore we tested Oases *k*-25 and two variations of multiple *k*-mer assembly: an additive assembly by pooling (Oases-P) as described by [28], and a merged assembly using the Oases-merge pipeline (Oases-M). Only transfrags above 100 bp were kept for downstream analysis.

### Redundancy check

A common attribute of *de novo* transcriptome assemblies is sequence redundancy. Using in-house PERL or PYTHON with suffix array scripts, we filtered for 100% identical copies and subsequences (*k*-mer) in both, forward and reverse directions. To compare our filtering approach with a typically applied post-assembly clustering step, we used CD-HIT-EST [29] with the following parameters: -M 0 -T 20 -g 0 -c 1.0 - b 1 -aL 1.0 -aS 1.0 -n 10 -d 0 -p 1 (duplicate removal, +\-) and -M 0 -T 20 -g 0 -c 1.0 -b 1 -aS 1.0 -n 10 -d 0 -p 1 (substring removal, +/+)*.* In addition, we evaluated the redundancy in each assembly using CD-HIT-EST as describe by [6].

### Coding potential assessment and conceptual translation

Assembled transcripts were evaluated for protein-coding attributes using PORTRAIT [30]. We corrected PORTRAIT to run ANGLE [31] in 6 frames, since the biological orientation of transfrags from non-strand specific libraries cannot be readily ascertained. The predicted open reading frame (ORF) with the highest dynamic programming score was chosen for conceptual translation into protein sequence using the standard codon usage table. Transfrags without an ORF were classified as orphan in this study. We note that they can be evaluated for coding capability through the protein-independent model of PORTRAIT.

### Transfrag annotation

We assigned protein domains to the predicted protein sequences using HMMER version 3.0 [32] with the manually curated protein profile Hidden Markov Models from Pfam (release 26.0, ftp://ftp.sanger.ac.uk). We then applied MultiPfam2go [33] to explore co-occurrence relationships between the domains of each protein and assigned functional labels (gene ontology terms) if the underlying domain architectures predicted protein function*.*

To mimic annotation of non-model organisms, we generated a BLAST-able database of UniProt Knowledgebase (FUNGI) release 2013_02 (The UniProt Consortium: http://www.uniprot.org/), excluding *N. crassa* sequences. We screened for highly significant BLASTX hits (max E-value 1e-10) using the NCBI BLAST package (version 2.2.25) and identified the top hit (lowest E-value, best scoring HSP covers minimum 25% of the hit) using custom PERL scripts.

### Validating *bona fide* transfrags by mapping to reference genome and predicted CDS

The *bona fide* transfrags were aligned to the reference CDS with BLAT v. 34 [34] to assess the integrity of assembly-derived transcripts. BLAT alignment in sim4 format were generate under intron restriction (-fastMap) with -minScore = 30 and post-alignment processing were performed through a series of custom PERL scripts.

Genome-base clustering was performed to assess gene space coverage by aligning *bona fide* transfrags to *N. crassa* reference genome with GMAP 2013-09-30.v2 [35]. The introns for *N. crassa* were obtained using Ensembl API [36] from http://fungi.ensembl.org release-17 to compute the maximum total length of intron per gene. Information about intron length statistics in fungi were obtain as described by [37] to parameterize transfrag and CDS alignment to the genome: min-intron length = 20, max-intron length = 2000, total length = 5904. The known gene loci are compared to the loci of aligned transfrags in a pair-wise manner using in-house PERL scripts to avoid building cluster chains [3]. Transfrags that do not overlap with CDS are clustered using Bedtools [38]. We aligned sequences belonging to the 5′ and 3′ untranslated regions of predicted genes and the Rfam family of predicted genes to the *N. crassa* genome under absolute condition of no introns using GMAP with a threshold of 95% coverage and 95% identity. The loci of these high-scoring alignments were compared to those of transfrags that did not overlap with CDS.

## Results

### *De novo* assembly and filtering *N. crassa* transfrags

The number of reads before and after quality filtering is shown in Table 1. Of the ~62 million reads that were processed, 82.5% survived quality trimming and were retained for subsequent *de novo* assembly.

**Table 1:** **Quality trimming statistics of** ***N. crassa*** **RNA-Seq data**

**Attributes**	**Raw reads**	**Processed reads pairs**	**Processed singletons**
Total read	31,301,048	24,390,689	2,849,486
Length, mean (min-max)	76 (76, 76)	72 (36, 76)	64 (36, 76)

A summary on assembly statistics for all four assembly methods is shown in Table 2. When comparing the two single *k*-mer assembly approaches (Trinity and Oases-25), we see that Trinity produced twice as many transfrags as Oases-25, but at much shorter transfrags lengths. These two assemblies had very little redundant transfrags compared to multiple *k*-mer assemblies. Multiple *k*-mer assemblies produced a much higher number of transfrags than single *k*-mer assemblies, but 38% - 56% were redundant. The median transfrag lengths for these assemblies were seven-fold greater than for the Trinity assembly. To compare our filtering procedure (in PERL or PYTHON scripts) with a typically applied post-assembly clustering method, we used CD-HIT-EST and generated a non-redundant assembly at 100% global identity. At these settings, our filtering method produced comparable results.

**Table 2:** **Attributes of** ***N. crassa*** **assemblies produced with different filtering approaches**

**Assembly**	**№ of TF (transfrags)**	**№ of unique TF (UTF)**	**Median unique TF length**	**% redundant TF PERL**	**% redundant TF CD-HIT-EST**
Trinity	35720	35578	240	0.4	0.4
Oases-25	19406	19193	1426	1.09	0.97
Oases-M	73215	45134	1839	38.35	38.35
Oases-P	138716	61293	1749	55.81	55.51

**Figure 2:**
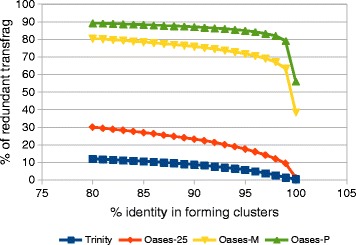
**An assessment of redundancy in various assemblies using CD-HIT-EST.** Comparing the fraction of redundant transfrags across all assemblies at various identity thresholds (80-100%) in creating clusters with CD-HIT-EST.

Typically, CD-HIT-EST is used at settings below 100% identity. The fraction of redundant transfrags at various identity thresholds for our *N. crassa* assemblies is shown in Figure 2. For the Oases-P assembly, at 80% identity nearly 90% of the transfrags are considered redundant by CD-HIT-EST, ie these transfrags can be incorporated into fewer clusters. This represents nearly 46,000 transfrags that are lost for downstream analysis when a representative transfrag is selected for a cluster as compared to clustering at 100% identity.

### Selecting *bona fide* transfrag and their functional annotation

Each non-redundant assembly was separated into two categories: *bona fide* (coding with predicted ORF) and orphan (non-coding, coding without ORF); numbers are displayed in Table 3. In Trinity, the proportion of orphan transfrags was higher (60%) than the proportion of *bona fide* transfrags. Trinity also produced a considerably higher number of orphan tranfrags than any of the three Oases assemblies. As a result, the number of *bona fide* transfrags was very similar for the two single *k*-mer assemblies, and Oases-P generated the highest number of *bona fide* transfrags.

**Table 3:** **Classification and annotation of the non-redundant** ***N. crassa*** **transfrags**

**Assembly**	**№ of UTF**	**№ of orphan UTF**	**№ of** ***bona fide*** **UTF**	**№ of orphan UTF with blast hit**	**№ of** ***bona fide*** **UTF with blast hit**	**№ of** ***bona fide*** **UTF with MultiPfam2go**
Trinity	35578	20772	14806	266 (1.3%)	10320 (70%)	6523 (44%)
Oases-25	19193	5359	13834	160 (3%)	11438 (83%)	6944 (50.2%)
Oases-M	45134	7453	37681	412 (6%)	31311 (83.1%)	18173 (48.2%)
Oases-P	61293	10848	50445	646 (6%)	41383 (82%)	24393 (48.4%)

**Figure 3:**
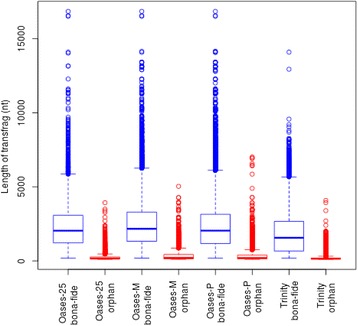
**Distribution of transfrag length for various assemblies.** *Bona fide* transfrags (blue): transfrag with coding potential and predicted CDS; orphan (red): transfrags with no coding potential or coding potential but no predicted CDS.

Figure 3 shows the distribution of transfrag lengths between *bona fide* and orphans transfrags. Orphan transfrags were generally much shorter than *bona fide* transfrags. For the *bona fide* transfrags of the three Oases assemblies, the median transfrag length (~2 kb) and the distributions are very similar. We note that the Oases assemblies had a considerable number of *bona fide* transfrags that were substantially longer than 10 kb. The median transfrag length of *bona fide* transfrags assembled using Trinity was 1.5 kb, and only a few of them were longer than 7.5 kb.

Non-redundant assemblies were annotated using BLAST and MultiPfam2go (Table 3). We note that in all assemblies only a small proportion of orphan transfrags had a BLAST match. Despite the highest number of orphan transfrags, Trinity had the least number of BLAST hits to transfrags in this category. In contrast, at least 70% of *bona fide* transfrags from all assemblies had a BLAST hit. This represented over 94% of cumulative BLASTx retrievable hit of the unfiltered assembly (Additional file 1). This number is higher than the ones typically reported in studies on *de novo* assembled transcriptomes [12,39]. In addition, *bona fide* transfrags were annotated with MultiPfam2go. The fraction of transfrags that could be associated with gene ontology terms ranged from 33%-50%, which is also high for domain based annotation [33].

### Assessing transfrag integrity and gene coverage

To evaluate the number of predicted genes represented by the *bona fide* transfrags, we aligned them to the predicted coding sequences (CDS) as well as to the genome of *N. crassa* (Table 4). Between 80% and 90% of the *bona fide* tranfrags mapped to both datasets at high stringency. Although the numbers of *bona fide* transfrags between single and multiple *k*-mer assemblies is very different, the number of identified genes is very similar. Most strikingly, Trinity identified the same number of predicted genes and putative unknown *N. crassa* gene loci as Oases-P, independent of the dataset and the alignment thresholds. As a result, the number of *bona fide* transfrags per gene is lower in single *k*-mer versus multiple *k*-mer assemblies. Orphan transfrags that mapped at the same stringency represented 15-40% of the known gene loci (Table 4), but ~ 90% were already identified by the longer *bona fide* categories. Unmapped transfrags mapped to multiple location and some were chimeric. The number of loci occupied by orphan transfrags ranged from 2,752 - 8,501. A look into the biological relevance of these loci revealed that they represent intronic, 5′ and 3′ untranslated regions and genes of the RFAM Family (Additional file 2).

**Table 4:** **Summary of** ***bona fide*** † and orphan* transfrags integrity and validity

**Assembly**	**№ of** ***bona fide*** **UTF**	**№ of chimeras in unmapped transfrags ζ**	**Alignment of TF to reference genes (CDS)**				**Alignment of TF to reference genome**	
			**№ of TF Cov 50%, ID 50%**	**№ of reference unigenes**	**№ of TF Cov 90%, ID 90%**	**№ of reference unigenes**	**№ of TF uniquely mapped**	**№ of** ***N. crassa*** **genes identified by TF**
Trinity^†^	14806	282	9879	6263	2593	1609	13339	7915
Oases-25^†^	13834	469	8653	5699	1086	784	11679	7345
Oases-M^†^	37681	3293	22080	5991	2751	1009	27931	7787
Oases-P^†^	50445	3417	30278	6179	4115	1249	39693	7906
Trinity*	20772	49	5016	1887	4063	1543	18553	3875
Oases-25*	5359	22	1292	844	1008	679	4918	1287
Oases-M*	7453	268	1512	916	976	653	6148	1483
Oases-P*	10848	247	2355	1234	1458	882	9203	1919

^†^Bona fide: transfrags with coding potential and predicted CDS.

*Orphan: transfrags with no coding potential or with coding potential but no predicted CDS.

ζ Possible chimera with a distinct breakpoint.

### Selecting *bona fide* assembly-derived transcripts in other species

We also verified the suitability of the IFRAT pipeline for selecting reconstructed transcripts in non-model organisms. The analysis results for unique transfrags longer than 100 bp from each draft assembly are show in Table 5. We predicted that up to 70% of the published transcriptome could be categorized as orphan transfrags. As before, the percentage of orphan transfrags with a BLAST hit was relatively low. In contrast, the proportion of *bona fide* transfrags with significant BLAST matches was often higher than in the unfiltered draft assemblies.

**Table 5:** **Allocation of BLASTX hits between** ***bona fide*** **and orphan transfrags inferred with IFRAT**

**Organism**	**№ of TF in publication**	**№ of TF with hit in publication**	**№ of UTF > = 100**	**№ of orphan UTF**	**№ of orphan UTF with blast hit**	**№ of bona fide UTF**	**№ of bona fide with blast hit**
*Hydra vulgaris*	48909	17587 (36%)	44484	9806 (22%)	1086 (11.1%)	34717	15310 (44.1%)
*Radix balthica*	41590	7347 (17.7%)	38790	26846 (69%)	1360 (5.1%)	11944	6723 (56.3%)
*Alipes grandidieri*	66199	16688 (25.2%)	66297	31355 (47%)	1809 (5.8%)	34942	12253 (35.1%)
*Cerebratulus marginatus*	80865	11062 (13.7%)	81021	46345 (57%)	782 (1.7%)	34676	9995 (28.8%)
*Chiton olivaceus*	93879	24495 (26.1%)	93885	52461 (56%)	1692 (3.2%)	41424	11001 (26.6%)
*Crella elegans*	31703	13984 (44.1%)	31172	10930 (35%)	1364 (12.5%)	20242	7439 (36.8%)
*Hormogaster samnitica*	90928	25681 (28.2%)	90928	41271 (45%)	1003 (2.4%)	49657	15392 (31%)
*Fagopyrum tataricum*	25041	19072 (76.1%)	25040	5747 (23%)	1909 (33.2%)	19294	16326 (84.6%)

## Discussion

Single *k*-mer assemblies of transcriptomes are considered incomplete because a short *k*-mer result in a highly diverse but also fragmented and redundant assembly, while a long *k*-mer provides a more contiguous assembly but misses poorly expressed transcripts [40]. To account for this problem the multiple *k*-mer transcriptome assembly approach was introduced [28,41]. However, the number of sequences generated in this way exceeds by far the number of protein coding genes likely to exist in the respective organism [42], making identification of genuine transfrags a major challenge for downstream analysis. To reduce redundancy, clustering or merging methods are currently being applied [4,27,43]. Yet, these methods rely on common word heuristics, ignoring the biological nature of assembled transcripts [44]. Therefore, reference-free clustering tends to mis-assign transfrags to biologically unrelated clusters [45] which leads to loss of unique functional annotations [6] and creation of chimeric transcripts [5].

Here, we propose IFRAT, a workflow that allows selection of unique *bona fide* transfrags (with CDS and coding potential) without clustering; and introduce domain co-occurrence analysis as means of tranfsrag assembly verification. IFRAT filters unique transfrags by removing exact duplicates, including identical forward and reverse nucleotide subsequences. IFRAT filtering removes slightly more transfrags than CD-HIT-EST at 100% identity because this program does not properly process transfrags containing N’s (author’s personal communication). Our results suggest that single *k*-mer assemblies may not need this filtering step since the proportion of redundant transfrags in the Trinity and Oases-25 datasets were only about 1%. In contrast, redundancy filtering is particularly important in multiple *k*-mer assemblies, considering that nearly half the transfrags in the Oases-M and Oases-P datasets were exact copies or substrings of other transfrags. It is unknown at what percent identity clustering results in significant loss of unique functional annotations. However, as suggested by our analysis, clustering without biological insight should be handled with caution because at 99% identity a significant subset of potentially unique transfrags is removed by CD-HIT-EST.

After filtering, IFRAT classifies the sequences into *bona fide* and orphan transfrags based on CDS prediction and coding potential. Our subsequent BLAST analysis corroborated this categorization, since 70-80% of *bona fide* transfrags had significant BLAST matches while this was true for only 1-6% of orphan tranfrags. We note that the median length of *bona fide* transfrags ranged from 1.5kb (Trinity) to 2kb (Oases), which is consistent with the average coding sequence length in fungi [46] while most of the orphan transfrags were short (med. 147-198 bp). However, our results confirmed previous findings that length is not the only indicator of coding potential [47] and ‘non-blastable’ transfrags [20], since 6-26% of the orphan transfrags with BLAST matches were less than 200 bp long.

All four assembly methods produced high quality datasets, as 76-90% of the transfrags mapped to the genome and the predicted CDS of *N. crassa* at high identity and coverage*. Bona fide* transfrags represented approximately 73% of the 10,785 known gene loci in the *N. crassa* genome. In addition, they indicated the existence of 715-1168 unknown potentially coding gene locations. Orphan transfrags also mapped to known gene locations, but most of these locations were represented by longer *bona fide* transfrags. These orphan transfrags may represent biologically interesting data, such as truncated assemblies (e.g. rare exons, poorly expressed genes, transcript with under-sampled regions), or immature mRNA with intronic regions and long UTRs for which coding potential could not be predicted [20,48,49]. Orphan transfrags that mapped to non-coding regions of the genomes represented ribosomal and non-coding RNA [50], and may be of interest. In any case, it is advisable to verify the correct assembly of orphan transfrags and remove mis-assemblies using a suitable reference dataset, such as a reference genome or EST collection. We integrated multi-domain co-occurrence architecture [33] to complement BLAST annotation. This avoids non-specific annotation of promiscuous domains resulting from truncated transfrags. Between 44% and 50% of the *bona fide* tranfrag peptides from *N. crassa* were assigned at least one GO term. Using IFRAT, we improved annotation coverage of published transcriptome datasets from non-model organisms. The choice of database and to a larger extend the coverage filter threshold accounts for small differences in the number of BLAST hits between *bona fide* transfrags and unfiltered assemblies. We attribute this high annotation coverage to the error tolerant CDS prediction [31] and selection of longer proteins with coding potential by IFRAT.

IFRAT is able to select *bona fide* transfrags irrespective of the assembler or assembly method used. Profound differences between transcriptome assemblers and assembly methods have been elaborately dealt with elsewhere [4,41,51,52]. We note however that Trinity performed very similar to Oases-P in identifying CDS and known gene loci, requiring substantially less computational resources. Other technical limitations, such as runtime and data-size, may influence the choice of one assembler over the other [53]. Since many more transfrags were produced by the multiple *k*-mer assemblies that identified a comparable number of gene loci, they may be suitable for studies with interest on splice variants.

## Conclusion

We have proposed a framework for post-assembly analysis of transcriptome assembly that is flexible enough to accommodate sequencing error and frame-shifts and that does not rely on the availability of a reference genome. Through this, a catalogue of reliable protein coding transfrags is established that represents a reference transcriptome. The method described herein is potentially applicable not only to assemblies of transcribed fragments generated with Trinity or Oases but also to assemblies produced by de Bruijn graph assemblers where no reliable sequenced genome is available, as demonstrated with the published datasets. Our framework performs well in segregating functionally relevant transcripts. We note that the main challenge remains the quality of assembly-derived-transcripts, which is undermined by incorporation of non-coding fragments that reduces the coding potential signal. One possible avenue for improvement is to screen RNA-seq reads for non-coding transcribed fragments prior to *de novo* assembly.

## Additional files

Additional file 1:
**Distribution of BLASTx hits between** ***bona fide*** **and orphan transfrags.** The *bona fide* transfrags are enriched with sequences that have a potential BLAST hit (34k). Additional file 2:
**Number of orphan transfrags that overlap with genic features and non-protein coding genes (33k).**
