# Supplementary figures and images for: Inferring bona fide transfrags in RNA-Seq derived-transcriptome assemblies of non-model organisms
Source: BMC Bioinformatics. 2015 Feb 21;16(1):58. doi: 10.1186/s12859-015-0492-5 (PMC4344733; doi:10.1186/s12859-015-0492-5)

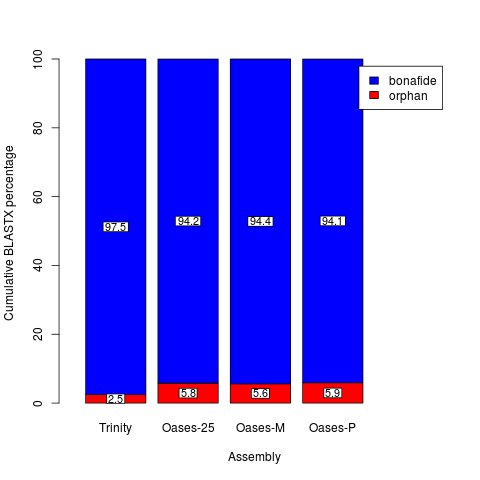

Supplement: Additional file 1: — Distribution of BLASTx hits between bona fide and orphan transfrags. The bona fide transfrags are enriched with sequences that have a potential BLAST hit (34k). [file 12859_2015_492_MOESM1_ESM.docx]
