# Supplementary material for: Inferring bona fide transfrags in RNA-Seq derived-transcriptome assemblies of non-model organisms
Source: BMC Bioinformatics. 2015 Feb 21;16(1):58. doi: 10.1186/s12859-015-0492-5 (PMC4344733; doi:10.1186/s12859-015-0492-5)
Supplement: Additional file 2: — Number of orphan transfrags that overlap with genic features and non-protein coding genes (33k). [file 12859_2015_492_MOESM2_ESM.doc]

| Assembly | № UTF orphans | № UTF orphans matching | | | |
| --- | --- | --- | --- | --- | --- |
|  |  | 5’-UTR | 3’-UTR | introns | Non-protein coding genes |
| Trinity | 20772 | 257 | 248 | 3321 | 20 |
| Oases-25 | 5359 | 93 | 99 | 499 | 11 |
| Oases-M | 7453 | 246 | 227 | 721 | 18 |
| Oases-P | 10848 | 513 | 609 | 1087 | 28 |

Number of orphan transfrags that overlap with genic features and non-protein coding genes
